# Supplementary material for: The Association between Seafood Intake and Fecundability: Analysis from Two Prospective Studies
Source: Nutrients. 2020 Jul 29;12(8):2276. doi: 10.3390/nu12082276 (PMC7469023; doi:10.3390/nu12082276)
Supplement: Supplementary file 1 [file nutrients-12-02276-s001.pdf]

Supplementary materials

Supplemental Table S1. Questions on seafood intake from food frequency questionnaires in each cohort.

Snart Foraeldre cohort:

Over the past year, how often, on average, have you eaten fish products?

	Per month			Per week			Per day		
Number of bread slices with this topping	None	≤1	2-3	1-2	3-4	5-6	1	2-3	≥4
Pickled or smoked fish (e.g., herring, salmon or mackerel in tomato sauce)									
Fried fish (e.g., fish filet or fish ball)									
Shrimp, mussels, crabs and the like									
Tuna (e.g., tuna in water, oil or tomato)									
Mayonnaise salad (e.g., tuna, mackerel or shrimp salad)									
Cod roe									

Over the past year, on average, how often had you eaten fish or seafood dishes? Consider all meals. However, here the number of bread slices is not considered

	Per month			Per week			Per day
Fish or shellfish	None	≤1	2-3	1-2	3-4	5-6	≥1

Over the past year, on average, how often have you eaten fish or seafood dishes?

	Per month			Per week			Per day
Number of dishes	None	≤1	2-3	1-2	3-4	5-6	≥1
Lean fish such as cod, pollock, plaice							
Oily fish such as salmon, herring, or mackerel							
Seafood (e.g., shrimp, crayfish, lobster tails)							
Other fish dishes, such as fish cakes or fish lasagna							
Sushi							

Also other lean fish such as tuna, flounder, garfish, turbot, haddock, hake, pangasius. Other oily fish such as trout, eel, sprat, lumpfish and halibut. Please think about the total number of meals you eat. However, spreads on bread and vegetables as an accompaniment to meat and fish are not counted here.

Over the past year, on average, how often have you eaten the dishes listed below?

	Per month			Per week			Per day
Number of dishes	None	≤1	2-3	1-2	3-4	5-6	≥1
Food pie with or without meat or fish (e.g., spinach pie or leek pie)							
Other soups with or without meat or fish (e.g., minestrone, potato or asparagus soup)							

Over the past year, on average, how often do you think you have eaten the below raw vegetables in a salad?

	Per month			Per week			Per day	
Number of meals	None	≤1	2-3	1-2	3-4	5-6	1	≥2
Green salad with fish, with or without other fillings								

PRESTO cohort (via the National Cancer Institute's Dietary History Questionnaire II):

Over the past 12 months…

1. How often did you eat **fried shellfish** (such as crab, lobster, shrimp)?
2. How often did you eat **shellfish** (such as crab, lobster, shrimp) **that was NOT FRIED**?
3. How often did you eat **salmon, fresh tuna, or trout**?
4. How often did you eat **canned tuna** (including in salads, sandwiches, or casseroles)?
5. How often did you eat **fish sticks** or other **fried fish** (not including shellfish)?
6. How often did you eat **other fish that was NOT FRIED** (not including shellfish)?

☐ NEVER

☐ 1–6 times per year
☐ 7–11 times per year
☐ 1 time per month
☐ 2–3 times per month
☐ 1 time per week
☐ 2 times per week
☐ 3–4 times per week
☐ 5–6 times per week
☐ 1 time per day
☐ 2 or more times per day

Supplementary Table S2. Association between seafood intake and fecundability, stratified by age at baseline.

	Snart Foraeldre				PRESTO				Snart Foraeldre				PRESTO			
	No. of pregs	No. of cycles	FR[a]	95% CI[a]	No. of pregs	No. of cycles	FR[a]	95% CI[a]	No. of pregs	No. of cycles	FR[a]	95% CI[a]	No. of pregs	No. of cycles	FR[a]	95% CI[a]
	Age <30 years								Age ≥30 years							
Total seafood, g/week																
<50	122	698	1.00	Ref	765	4954	1.00	Ref	79	372	1.00	Ref	660	4893	1.00	Ref
50-99	215	1084	1.04	0.84-1.30	369	2231	1.02	0.91-1.14	141	760	0.81	0.63-1.05	461	2783	1.10	0.99-1.23
100-199	433	2324	0.95	0.78-1.15	282	1604	1.04	0.92-1.18	312	1656	0.85	0.68-1.07	406	2521	1.05	0.93-1.18
≥200	266	1494	0.87	0.70-1.09	129	770	1.00	0.84-1.19	250	1221	0.98	0.76-1.25	195	1320	1.01	0.87-1.17
Shellfish, g/week																
None	63	451	1.00	Ref	753	4767	1.00	Ref	44	204	1.00	Ref	635	4580	1.00	Ref
1-9	502	2553	1.45	1.13-1.84	329	1873	1.04	0.93-1.17	378	1919	0.94	0.71-1.25	395	2598	1.01	0.90-1.13
10-49	436	2417	1.33	1.03-1.71	354	2214	0.98	0.87-1.10	332	1764	0.93	0.69-1.26	536	3287	1.06	0.95-1.18
≥50	35	179	1.29	0.87-1.90	109	705	0.94	0.78-1.13	28	122	1.04	0.66-1.64	156	1052	0.99	0.84-1.16
Unfried shellfish, g/week [b]																
None					812	5155	1.00	Ref					682	4897	1.00	Ref
1-9					378	2163	1.04	0.93-1.16					503	3286	1.01	0.91-1.12
10-19					146	937	0.96	0.82-1.13					228	1309	1.11	0.97-1.28
≥20					209	1304	0.97	0.84-1.12					309	2025	0.99	0.87-1.12
Fried shellfish, g/week [b]																
None					1137	6953	1.00	Ref					1169	7811	1.00	Ref
1-4					223	1375	0.97	0.85-1.10					335	2173	0.98	0.88-1.10
5-9					156	967	1.00	0.86-1.17					185	1278	0.99	0.86-1.14
≥10					29	264	0.73	0.52-1.03					33	255	0.81	0.58-1.12

[a] Adjusted for energy intake, age, BMI, smoking status, alcohol intake, physical activity, last form of contraception, marital status, intercourse frequency, use of methods to improve pregnancy chances, daily multivitamin use, use of fish oil supplements, education, income, healthy diet indices, sugar-sweetened beverage intake, and parity.
[b] Variables not available in Snart Foraeldre.

Supplementary Table S3. Association between seafood intake and fecundability, stratified by body mass index.

	Snart Foraeldre				PRESTO				Snart Foraeldre				PRESTO			
	No. pregs	No. cycles	FR[a]	95% CI[a]	No. pregs	No. cycles	FR[a]	95% CI[a]	No. pregs	No. cycles	FR[a]	95% CI[a]	No. pregs	No. cycles	FR[a]	95% CI[a]
	Body mass index <25 kg/m^2								Body mass index ≥25 kg/m^2							
Total seafood, grams/week																
<50	132	670	1.00	Ref	714	4100	1.00	Ref	69	400	1.00	Ref	711	5747	1.00	Ref
50-99	247	1252	0.92	0.76-1.12	455	2503	1.04	0.93-1.15	109	592	1.06	0.80-1.40	375	2511	1.08	0.96-1.21
100-199	525	2751	0.90	0.76-1.07	394	2057	1.07	0.96-1.20	220	1229	1.00	0.77-1.29	294	2068	1.02	0.89-1.16
≥200	382	1957	0.92	0.75-1.11	178	1067	0.99	0.85-1.15	134	758	0.96	0.71-1.29	146	1023	1.06	0.89-1.25
Shellfish, grams/week																
None	76	427	1.00	Ref	721	4160	1.00	Ref	31	228	1.00	Ref	667	5187	1.00	Ref
1-9	631	3102	1.15	0.92-1.43	391	2019	1.05	0.94-1.17	249	1370	1.39	0.97-1.99	333	2452	0.95	0.84-1.07
10-49	541	2923	1.09	0.86-1.37	478	2660	1.03	0.93-1.15	227	1258	1.33	0.93-1.91	412	2841	1.01	0.90-1.13
≥50	38	178	1.17	0.82-1.67	151	888	0.99	0.84-1.16	25	123	1.21	0.74-1.99	114	869	0.95	0.79-1.15
Unfried shellfish, grams/week																
None					772	4454	1.00	Ref					722	5598	1.00	Ref
1-9					470	2498	1.05	0.95-1.17					411	2951	0.95	0.85-1.07
10-19					202	1090	1.08	0.94-1.25					172	1156	1.02	0.88-1.19
≥20					297	1685	1.02	0.90-1.16					221	1644	0.95	0.82-1.09
Fried shellfish, grams/week																
None					1249	6959	1.00	Ref					1057	7805	1.00	Ref
1-4					283	1506	0.99	0.89-1.11					275	2042	0.96	0.85-1.09
5-9					176	1002	0.97	0.84-1.12					165	1243	1.02	0.88-1.19
≥10					33	260	0.74	0.54-1.02					29	259	0.81	0.57-1.15

[a] Adjusted for energy intake, age, BMI, smoking status, alcohol intake, physical activity, last form of contraception, marital status, intercourse frequency, use of methods to improve pregnancy chances, multivitamin use, use of fish oil supplements, education, income, healthy diet indices, sugar-sweetened beverage intake, and parity.

Supplementary Table S4. Association between seafood intake and fecundability, stratified by pregnancy attempt time at cohort entry.

	Snart Foraeldre				PRESTO				Snart Foraeldre				PRESTO			
	No. of pregs	No. of cycles	FR[a]	95% CI[a]	No. of pregs	No. of cycles	FR[a]	95% CI[a]	No. of pregs	No. of cycles	FR[a]	95% CI[a]	No. of pregs	No. of cycles	FR[a]	95% CI[a]
	Attempt time at entry <3 cycles								Attempt time at entry ≥3 cycles							
Total seafood, g/week																
<50	124	649	1.00	Ref	1070	6732	1.00	Ref	77	421	1.00	Ref	355	3115	1.00	Ref
50-99	238	1247	0.96	0.78-1.17	621	3479	1.03	0.94-1.13	118	597	1.14	0.86-1.52	209	1535	1.16	0.99-1.36
100-199	535	2637	0.98	0.82-1.17	535	2968	1.03	0.94-1.13	210	1343	0.92	0.71-1.19	153	1157	1.14	0.95-1.37
≥200	350	1778	0.95	0.78-1.17	241	1416	0.98	0.86-1.11	166	937	1.00	0.74-1.34	83	674	1.07	0.84-1.35
Shellfish, g/week																
None	70	428	1.00	Ref	1040	6393	1.00	Ref	37	227	1.00	Ref	348	2954	1.00	Ref
1-9	602	2902	1.27	1.01-1.60	543	3070	1.01	0.92-1.11	278	1570	1.13	0.82-1.57	181	1401	1.02	0.86-1.20
10-49	529	2764	1.19	0.94-1.51	683	3894	1.02	0.93-1.11	239	1417	1.13	0.81-1.59	207	1607	1.03	0.87-1.21
≥50	46	217	1.23	0.86-1.76	201	1238	0.95	0.83-1.09	17	84	1.26	0.70-2.24	64	519	1.00	0.77-1.30
Unfried shellfish, g/week [b]																
None					1123	6881	1.00	Ref					371	3171	1.00	Ref
1-9					658	3757	1.00	0.92-1.09					223	1692	1.06	0.90-1.24
10-19					287	1611	1.05	0.93-1.18					87	635	1.07	0.86-1.34
≥20					399	2346	0.98	0.88-1.08					119	983	0.98	0.80-1.20
Fried shellfish, g/week [b]																
None					1737	10123	1.00	Ref					569	4641	1.00	Ref
1-4					433	2562	0.99	0.90-1.08					125	986	0.93	0.78-1.12
5-9					252	1545	0.98	0.87-1.10					89	700	1.04	0.84-1.29
≥10					45	365	0.74	0.56-0.97					17	154	0.92	0.58-1.45

[a] Adjusted for energy intake, age, BMI, smoking status, alcohol intake, physical activity, last form of contraception, marital status, intercourse frequency, use of methods to improve pregnancy chances, daily multivitamin use, use of fish oil supplements, education, income, healthy diet indices, sugar-sweetened beverage intake, and parity.
[b] Variables not available in Snart Foraeldre.

Supplementary Table S5. Association between seafood intake, marine fatty acids, and fecundability, among non-consumers of fish oil supplements.

	Snart Foraeldre					PRESTO				
		No. of pregs	No. of cycles	FR[a]	95% CI[a]		No. of pregs	No. of cycles	FR[a]	95% CI[a]
Total seafood, g/week										
<50		163	895	1.00	Ref		1194	8318	1.00	Ref
50-99		288	1510	0.97	0.81-1.17		661	3891	1.07	0.98-1.16
100-199		615	3233	0.96	0.82-1.13		526	3365	0.99	0.90-1.09
≥200		414	2108	0.96	0.81-1.17		241	1560	0.98	0.86-1.11
Shellfish, g/week										
None		90	533	1.00	Ref		1150	7771	1.00	Ref
1-9		716	3623	1.22	1.00-1.50		584	3610	1.00	0.92-1.10
10-49		626	3367	1.17	0.94-1.44		687	4383	0.99	0.91-1.08
≥50		48	223	1.24	0.90-1.72		201	1370	0.92	0.80-1.06
Unfried shellfish, g/week[b]										
None							1243	8341	1.00	Ref
1-9							690	4398	0.98	0.90-1.07
10-19							286	1806	1.00	0.89-1.12
≥20							403	2589	0.96	0.86-1.06
Fried shellfish, g/week[b]										
None							1868	12034	1.00	Ref
1-4							417	2787	0.94	0.86-1.04
5-9							286	1864	1.00	0.89-1.12
≥10							51	449	0.75	0.58-0.97
Total marine fatty acids percentile										
<25th	<1.00	353	1959	1.00	Ref	<0.37	674	4555	1.00	Ref
25th-49th	1.00-1.83	379	1966	1.01	0.88-1.15	0.37-0.60	625	4601	0.88	0.80-0.97
50th-74th	1.84-2.91	397	2011	1.03	0.90-1.18	0.61-1.03	678	4094	1.00	0.90-1.10
75th-89th	2.92-4.30	212	1085	1.00	0.85-1.17	1.04-1.64	402	2284	1.02	0.91-1.14
≥90th	≥4.31	139	725	1.06	0.88-1.27	≥1.65	243	1600	0.89	0.78-1.02
EPA percentile										
<25th	<0.29	352	1929	1.00	Ref	<0.09	643	4624	1.00	Ref
25th-49th	0.29-0.52	381	2026	0.98	0.86-1.12	0.09-0.16	658	4535	0.99	0.90-1.10
50th-74th	0.53-0.82	393	1999	1.02	0.89-1.16	0.17-0.31	677	4126	1.07	0.97-1.18
75th-89th	0.83-1.19	212	1104	0.98	0.83-1.15	0.32-0.54	410	2282	1.09	0.97-1.22
≥90th	≥1.20	142	688	1.12	0.94-1.35	≥0.55	234	1567	0.94	0.82-1.08

DPA percentile										
<25th	<0.07	365	1985	1.00	Ref	<0.07	628	4405	1.00	Ref
25th-49th	0.07-1.13	365	1986	0.98	0.86-1.12	0.07	693	4347	1.06	0.95-1.19
50th-74th	1.14-0.21	395	1957	1.05	0.92-1.20	0.08-0.11	653	4400	1.03	0.91-1.18
75th-89th	0.22-0.33	214	1106	0.99	0.84-1.16	0.12-0.15	392	2487	1.01	0.90-1.13
≥90th	≥0.34	141	712	1.10	0.92-1.32	≥0.16	256	1495	1.05	0.92-1.20
DHA percentile										
<25th	<0.64	355	1967	1.00	Ref	<0.20	668	4573	1.00	Ref
25th-49th	0.64-1.17	377	1945	1.01	0.88-1.15	0.20-0.36	641	4629	0.90	0.81-0.99
50th-74th	1.18-1.86	397	2003	1.03	0.90-1.18	0.37-0.61	656	4078	0.98	0.89-1.08
75th-89th	1.87-2.78	211	1109	0.98	0.83-1.15	0.62-0.96	408	2230	1.06	0.95-1.19
≥90th	≥2.79	140	722	1.07	0.89-1.28	≥0.97	249	1624	0.91	0.79-1.04

REF = reference group. [a] Adjusted for energy intake, age, BMI, smoking status, alcohol intake, physical activity, last form of contraception, marital status, intercourse frequency, use of methods to improve pregnancy chances, daily multivitamin use, use of fish oil supplements, education, income, Healthy Eating Index score/NRD score, sugar-sweetened beverage intake, and parity. [b] Variables not available in Snart Foraeldre.
